# Pathogenic mechanisms and vaccine development for *Mycoplasma gallisepticum* in chickens

**DOI:** 10.3389/fmicb.2025.1741449

**Published:** 2026-01-12

**Authors:** Jiaxin Chen, Peng Liu, Ying Chen

**Affiliations:** 1 Department of Pharmacy, Affiliated Hengyang Hospital of Hunan Normal University & Hengyang Central Hospital, Hengyang, Hunan, China; 2 Institute of Pathogenic Biology, Basic Medical School, Hengyang Medical School, University of South China, Hengyang, Hunan, China

**Keywords:** Avian mycoplasma, *Mollicutes*, *Mycoplasma gallisepticum*, pathogenic mechanisms, vaccine

## Abstract

*Mycoplasma gallisepticum* (MG) is a significant avian pathogen responsible for chronic respiratory disease in chickens and infectious sinusitis in turkeys (*Meleagris gallopavo*). It infects the respiratory tract, leading to chronic inflammation and, in some cases, conditions such as synovitis and arthritis. MG causes substantial economic losses in the poultry industry due to reduced egg production, hatchability, meat quality, and increased mortality. The primary pathogenic mechanism involves immune dysregulation, enabling the bacterium to persist in the host and establish chronic infection. Key virulence factors include adhesins (e.g., GapA, CrmA, pMGA), variable surface lipoproteins (e.g., VlhA), and recently characterized molecules like the TatD nuclease. Current control measures include antibiotics, management adjustments, and vaccination. However, extensive use of broad-spectrum antibiotics like tetracyclines and macrolides has led to increased drug resistance. Although commercial vaccines (live attenuated, inactivated, and newer epitope-based and recombinant designs) are available, they often provide incomplete or inefficient protection. This review summarizes the current understanding of MG pathogenesis, highlights recent advances in vaccine development, and discusses the limitations and future directions for MG control.

## 1 Introduction

*Mollicutes*, encompassing four phylogenetic categories—*Spiroplasma, Hominis*, *Pneumoniae* and *Acholeplasma/Anaeroplasma/Phytoplasma* (Trachtenberg, 2005; Miyata and Hamaguchi, 2016a; Miyata and Hamaguchi, 2016b), impact the health of animals (Huang et al., 2019; Hu et al., 2022; He et al., 2024; Luo et al., 2021; Yu et al., 2020) and plants (You et al., 2024; Qiu et al., 2024). Having evolved from Gram-positive bacterial ancestors, *Mollicutes* have dispensed with their peptidoglycan layer and undergone genome reduction, resulting in a distinctive wall-less structure (Hu et al., 2022; Zhou et al., 2021; Gründel et al., 2015). *Mycoplasma gallisepticum* (MG) belongs to the class *Mollicutes* (Razin et al., 1998; Chen et al., 2025). Its lack of a cell wall makes it inherently resistant to beta-lactam antibiotics, and tetracyclines are commonly used for prevention (Hannan, 2000). Among the 25 known avian mycoplasmas, four are considered pathogenic: MG, *Mycoplasma synoviae* (MS), *Mycoplasma meleagridis* (MM), and *Mycoplasma iowae* (MI). MG and MS are the primary pathogens, listed by the World Organisation for Animal Health (OIE) (Wang H. et al., 2025). MG is a major cause of chronic respiratory disease in chickens (Feberwee et al., 2022; Wang Y. et al., 2025), colonizing the respiratory tract and potentially disseminating systemically (Ishfaq et al., 2021). Infection stimulates inflammatory cell activation and infiltration, leading to clinical signs such as sneezing and coughing (Zhang et al., 2020; Niu et al., 2020).

MG is recognized as one of the most significant avian pathogens from both clinical and economic perspectives for the global chicken and turkey farming industry (Marouf et al., 2022). The economic impact stems from its high prevalence and the substantial production losses it causes. Recent epidemiological studies continue to document its widespread nature. For instance, in Bangladesh, a molecular study (PCR) revealed an MG infection rate of 25.6% in turkeys (Rufai et al., 2025). A recent systematic review and meta-analysis (2022) estimated the global pooled molecular occurrence of MG in poultry to be 27.0%, highlighting its pervasive nature and substantial impact on production systems worldwide (Chaidez-Ibarra et al., 2022). Regional studies continue to document its significant presence; for instance, serological surveys in Egypt have reported infection rates of 10.9% in chicken flocks (El-Ashram et al., 2021). Such widespread infection leads to chronic respiratory disease in chickens, infectious sinusitis in turkeys, alongside reductions in weight gain, feed efficiency, and egg production, collectively resulting in severe economic losses for the poultry sector.

Mycoplasmas are considered model organisms for studying minimal cellular genomes, having evolved from low G+C Gram-positive bacteria through reductive evolution (Woese et al., 1980; Zhang et al., 2025; Yu et al., 2025; Li et al., 2025). The most notable genomic reduction is the loss of biosynthetic pathways, including those for cell wall synthesis (Chambaud et al., 2001; Yang X. et al., 2025; Yang Y. et al., 2025). The complete genome sequence of MG strain R_low_ (accession number: AE015450) has been determined, revealing a genome size of 996,422 bp with a G + C content of 31 mol% and 742 predicted coding sequences (CDSs) (Papazisi et al., 2003). The replication origin was identified based on sequence analysis of the dnaA gene region, featuring high AT content and repetitive sequences (Baker and Wickner, 1992). The VlhA gene family, comprising 43 genes distributed across five loci, accounts for approximately 10.4% of the genome and is implicated in antigenic variation (Papazisi et al., 2003; Markham et al., 1994). Additionally, 80 genes are predicted to encode lipoproteins, and 149 proteins contain multiple transmembrane domains, with several identified as potential virulence factors (Papazisi et al., 2003).

Current control strategies for MG include antimicrobial therapy, biosecurity measures, and vaccination. However, rising antibiotic resistance underscores the need for effective vaccines as a sustainable control tool (Bekő et al., 2019). Among these, the ts-11 strain, a temperature-sensitive mutant, is licensed in several countries, including the United States, Italy, and Australia (Kanci Condello et al., 2020). Nevertheless, existing vaccines, including live attenuated and inactivated types, often provide incomplete or temporary protection and may have drawbacks such as residual pathogenicity or interference with disease surveillance (Ishfaq et al., 2020). Given this persistent and considerable global burden, a thorough understanding of MG’s pathogenic mechanisms and the ongoing development of effective vaccines are critical for advancing poultry health and ensuring economic sustainability. This review aims to systematically summarize the pathogenic mechanisms of MG and the current status as well as future directions in vaccine development.

## 2 Pathogenic mechanisms

MG can be transmitted vertically from infected hens to their offspring through eggs (in ovo) and embryos (Roberts and McDaniel, 1967). This route of transmission is often a consequence of respiratory infection in hens, facilitated by the close anatomical proximity between the abdominal air sacs and the oviduct. The rate of vertical transmission varies widely depending on environmental conditions, individual bird factors, and the stage of infection, with peak transmission occurring during the acute phase of disease when respiratory MG loads are highest (Cobb, 2011). It is important to note that even some live attenuated vaccine strains, such as ts-11, retain the ability for vertical transmission (Armour and Ferguson-Noel, 2015).

**Figure 1:**
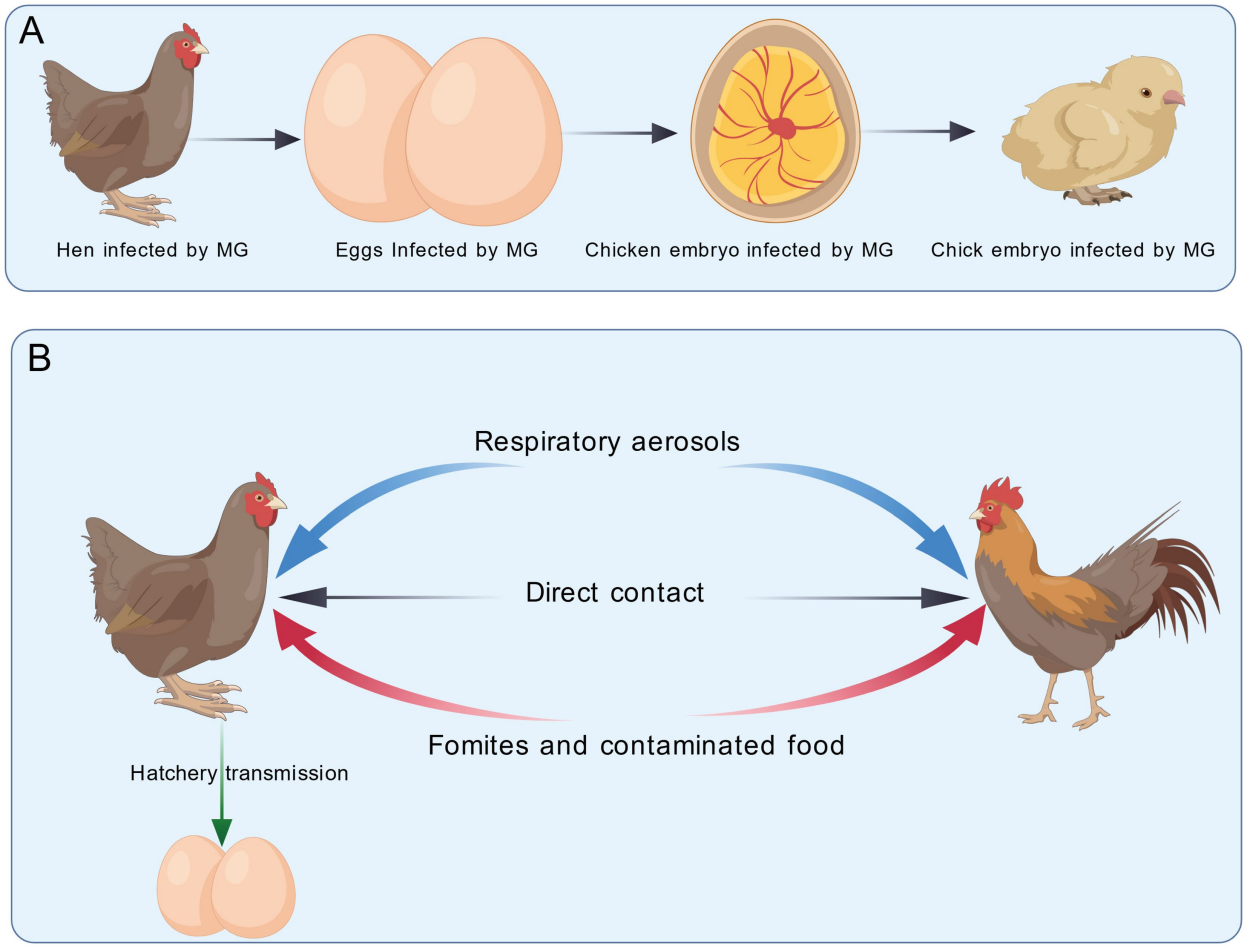
Schematic diagram of MG transmission routes. **(A)** Vertical transmission: MG is transmitted from infected hens to their offspring via eggs (in ovo) and embryos. **(B)** Horizontal transmission: MG spreads through respiratory aerosols, direct contact, fomites, contaminated food, and within the hatchery. This figure was created with BioGDP.com (Jiang et al., 2025).

Horizontal transmission of MG occurs through direct or indirect contact between birds. Direct transmission primarily takes place via respiratory aerosols and close contact with infected individuals. Once introduced into a susceptible flock, MG typically spreads rapidly (Xu et al., 2021). Indirect transmission can occur through multiple environmental routes: contaminated feed and shared feeders act as common fomites (Jiang et al., 2021), and hatchery transmission is possible through debris from broken infected eggs. The pathogen can survive on various surfaces, including feathers, egg contents, and human skin for 1–2 days, and on bird feeders for up to 1 day, facilitating its spread. Therefore, implementing strict biosecurity measures, including regular cleaning and disinfection, is crucial to interrupt the horizontal transmission cycle (Elliott et al., 2019; Mugunthan et al., 2023a) (Figure 1).

MG expresses adhesins that facilitate attachment and colonization of host epithelial cells (Distelhorst et al., 2017). The pathogen can evade the host immune system, leading to immune dysregulation, mitochondrial damage, and increased reactive oxygen species production (Chen et al., 2020). Mycoplasmas, including MG, can invade non-phagocytic cells and persist within phagocytes, contributing to chronic inflammation and ineffective clearance (Vogl et al., 2008; Much et al., 2002; Yueyue et al., 2022). Phenotypic variation in size and antigenicity aids MG in adapting to complex host environments (Noormohammadi, 2007). Studies using Hep-2 cells as a model have shown that mycoplasma internalization may be a key strategy for crossing mucosal barriers and evading host defenses (Buim et al., 2011).

MG colonizes its host mainly via the mucosal surfaces of the respiratory tract, causing air sacculitis within a few days, and disseminates throughout the body. This systemic infection is reflected by the high rate of MG reisolation from inner organs such as the liver, heart, spleen, or kidney and by its detection inside and at the surface of red blood cells of experimentally infected birds (Mugunthan et al., 2023a; Majumder and Silbart, 2016; Indiková et al., 2013). Key mechanisms for survival in the host include adhesion to host cells, antigenic variation, induction of apoptosis, and host cell damage (Beaudet et al., 2017). Lacking a cell wall, MG relies on membrane-anchored lipoproteins for host interaction, and it encodes a high proportion of putative lipoproteins compared to other prokaryotes (Chambaud et al., 1999).

### 2.1 Adhesion to host cells

**Figure 2:**
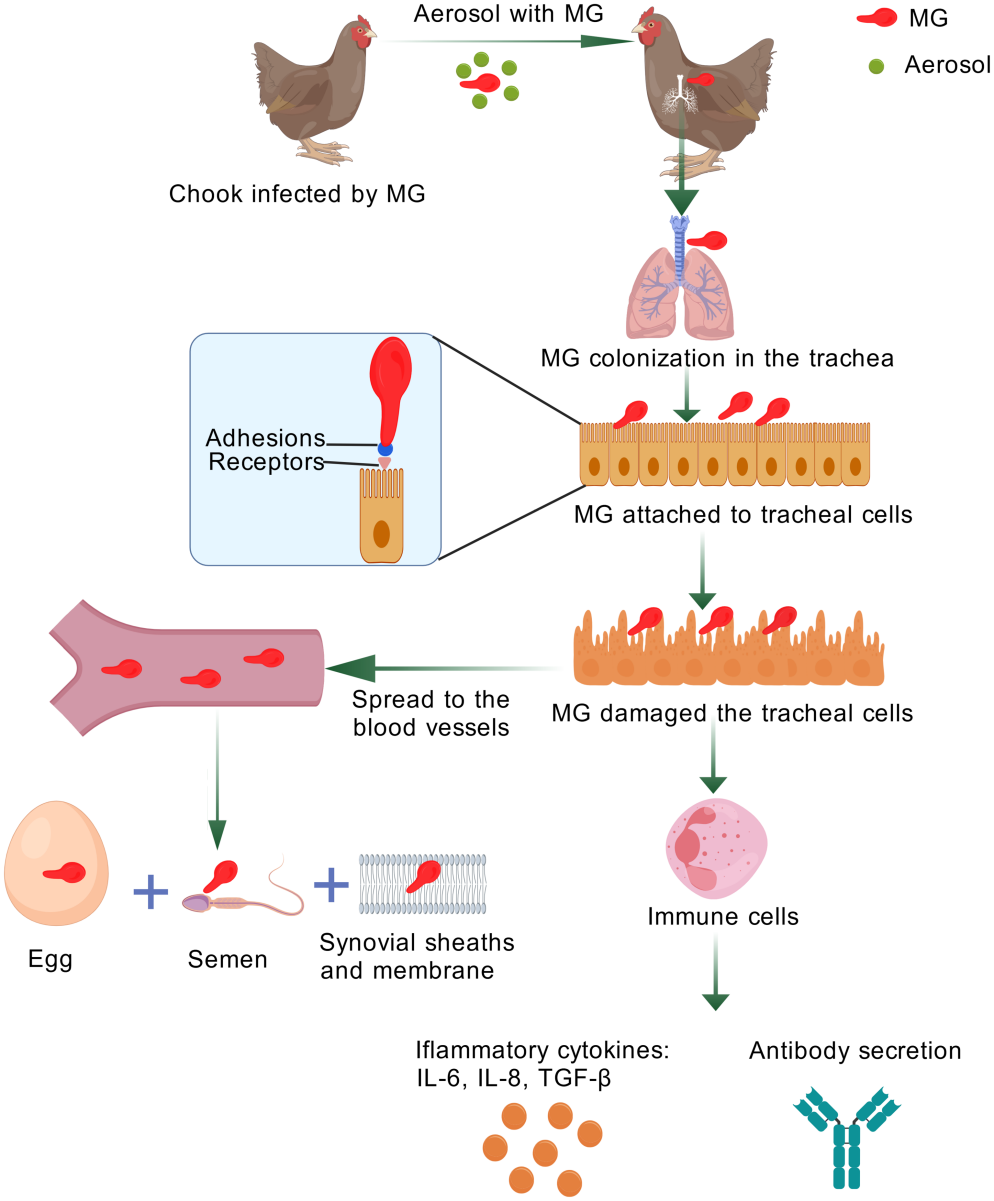
Pathogenic mechanism of MG. Following airway entry, MG adheres to bronchial epithelial cells via the key adhesins GapA and CrmA on its terminal tip. After establishing persistent colonization, MG can disseminate systemically to the joints and reproductive tract. Tracheal damage induces a B-cell response (antibody IgM/IgA) and CD4+/CD8 + T-cell infiltration. Colonization also triggers significant inflammation: MG lipoproteins activate TLR2, leading to NF-κB activation and the release of pro-inflammatory mediators (e.g., IL-1β, IL-8). This figure was created with BioGDP.com (Jiang et al., 2025).

MG and *Mycoplasma pneumoniae* possess specialized terminal structures that facilitate gliding motility and host cell attachment (Chen et al., 2025; Liao et al., 2021). Electron microscopy studies first associated the bleb structure of MG with adhesion, showing bacterial cells clustered around leukocytes at these sites. Gliding motility enables traversal of host physical barriers like ciliary activity and mucus layers (Bower et al., 2003). Adhesion to host epithelial cells is a critical first step in MG pathogenesis (Figure 2). The absence of a cell wall means MG lacks endotoxin and does not produce known exotoxins (Cole, 1991; Hu et al., 2016). Instead, surface lipoproteins are crucial for adhesion, invasion, and immune modulation. The MALFABC transporter in MG is involved in glycerol transport, enhancing colonization (Mahdizadeh et al., 2021). Major adhesion-related proteins include GapA, CrmA (Indiková et al., 2013; Papazisi et al., 2002), Mgc1/2 (Hnatow et al., 1998; Yiwen et al., 2021), PvpA (Mahdizadeh et al., 2021), an OsmC-like protein (MG1142) (Jenkins et al., 2007), and PDHA/PDHB (Qi et al., 2018). MG invades host cells, facilitating colonization (Vogl et al., 2008), and adheres to respiratory mucosa via membrane proteins such as GapA, PvpA, CrmA, pMGA1.2, and Mgc2/3. Attenuated strains lacking these adhesins fail to establish infection (Bashashati and Banani, 2020). pMGA1.2 plays a pivotal role in adhesion and invasion (Hu et al., 2016). During early infection, gga-miR-365-3p expression is upregulated, suppressing pMGA1.2, while SOCS5 expression increases later, enhancing pMGA1.2 expression and potentially promoting adhesion (Wang et al., 2022; Wang S. et al., 2025) (Table 1).

**Table 1:** Major adhesion related proteins of MG.

Protein	Characterization	Function in pathogenesis	References
GapA	Cytadhesin, part of the terminal organelle	Essential for attachment to host respiratory epithelium; co-expression with CrmA is critical for virulence.	Papazisi et al. (2000)
CrmA	Cytadhesin, part of the terminal organelle	Works synergistically with GapA for stable adhesion and gliding motility.	Indiková et al. (2013)
pMGA	Variable lipoprotein hemagglutinin, large multigene family	Mediates adhesion and is a key molecule for antigenic variation, enabling immune evasion.	Bencina (2002)
Mgc1	Cytadhesin	Homologous to *M. pneumoniae* P1 protein; involved in attachment.	Keeler et al. (1996)
Mgc2	Cytadhesin	Homologous to *M. pneumoniae* P30 protein; involved in attachment.	Hnatow et al. (1998)
PvpA	Variable surface protein	Involved in adhesion and may contribute to antigenic variation.	Boguslavsky et al. (2000)
OsmC-like (MG1142)	Surface-exposed protein	Binds heparin; potential role in adhesion to host cells or ECM.	Jenkins et al. (2007)
PDHA/PDHB	Pyruvate dehydrogenase subunits	Surface-localized; involved in cytoadherence; may link metabolic function to adhesion.	Qi et al. (2018)
Hlp3	Surface lipoprotein	Binds to fibronectin, facilitating adhesion to the ECM.	Mugunthan et al. (2023b)

Other surface proteins, including hemadsorption proteins GapA and CrmA, and variable membrane proteins (e.g., p30, p48, p50, p80), mediate cell attachment and invasion (Xu et al., 2021). MG and *M. pneumoniae* specifically bind sialoglycoprotein receptors on host cells (Banai et al., 1981), though not all mycoplasmas utilize sialic acid for attachment (Chen et al., 2025) (Table 1).

Adhesion to the extracellular matrix (ECM) is essential for tissue colonization (Duensing et al., 1999). ECM components like fibronectin and vitronectin act as molecular bridges, binding both pathogens and host cell receptors (Bower et al., 2003; Chaussee et al., 2000). MG utilizes ECM proteins as a secondary anchoring system (Limsatanun et al., 2022), and surface proteins such as PlpA and Hlp3 bind to fibronectin domains (Goh et al., 1998) (Table 1).

### 2.2 Immune evasion

MG exhibits adaptive strategies to evade the host immune system, establishing persistent infection. Firm attachment via adhesion molecules helps avoid rapid clearance by host defenses (Noormohammadi, 2007). MG infection triggers a strong inflammatory response (Figure 2), characterized by PAMP (Pathogen-Associated Molecular Pattern) (Yu et al., 2018) and DAMP (Damage-Associated Molecular Pattern) (Wang et al., 2024) presence, upregulated PRRs (Pattern Recognition Receptors) (Zou et al., 2020) and cytokine receptors (Zou et al., 2020), and recruitment of immune cells to the lamina propria, contributing to chronic infection (Gaunson et al., 2000; Lam, 2004; Gaunson et al., 2006). Despite its small genome, MG displays significant gene expression plasticity in response to environmental stress (Wang et al., 2022).

Antigenic variation is a major immune evasion strategy. The pMGA gene family allows MG to switch expressed adhesion proteins, generating antigenic variants (Wang et al., 2022). MG infection activates the JAK/STAT signaling pathway, which regulates immune responses to various pathogens (Yao et al., 2017). During infection, pro-inflammatory cytokines (e.g., TNF-*α*, IL-6) are upregulated (Figure 2). Modulation of gga-miR-365-3p and SOCS5 affects pMGA1.2 expression and inflammatory cytokine secretion, promoting bacterial survival (Wang et al., 2022). The VlhA gene family also mediates adhesion and immune escape (Orlov et al., 2018). Pathogenic MG isolates from wild birds exhibit VlhA gene variation (Matyushkina et al., 2016). Variable lipoprotein hemagglutinin (VlhA) is crucial for pathogenesis, with MSPA being a dominant antigen in MS that mediates adhesion (Rosengarten et al., 2000). MG alters its surface VlhA antigen repertoire during eukaryotic cell infection, upregulating defense proteins (Matyushkina et al., 2016). Single nucleotide polymorphisms (SNPs) in vlhA genes contribute to antigenic diversity, a key survival strategy (Markham et al., 1994; Rosengarten et al., 2000). MG also adapts its metabolism during infection; upregulation of NADH oxidase may facilitate ATP synthesis via pyruvate dehydrogenase, meeting increased energy demands (Matyushkina et al., 2016; Rosales et al., 2017). Heat shock proteins (HSPs), particularly surface-localized HSP60 (GroEL), promote pathogen adhesion and can induce host cell apoptosis and inflammation (Ensgraber and Loos, 1992; Kol et al., 1999).

### 2.3 Apoptosis and host cell damage

MG replicates in various cell types, including HeLa-229 cells, chicken embryo fibroblasts, and non-phagocytic cells like chicken erythrocytes (Vogl et al., 2008; Mahdizadeh et al., 2021). It disseminates to organs such as the spleen, heart, brain, and kidneys (You et al., 2006). Infection initiates with adhesion, mediated by proteins like GapA and CrmA (Papazisi et al., 2002; Qi et al., 2018; Seto and Miyata, 2003). Electron microscopy reveals chromatin condensation, mitochondrial swelling, and apoptotic vesicles in MG-infected cells, with inflammatory cell infiltration and tissue damage observed in thymic sections, indicating apoptosis (Li et al., 2019). MG-induced cell damage is linked to inflammation and oxidative stress. Infection disrupts pro-inflammatory cytokine balance (e.g., IL-6, IL-1β, IL-8, IL-10, IFN-*γ*, TNF-α), promoting leukocyte accumulation and inflammation (Figure 2). Oxidative stress exacerbates inflammation and apoptosis, contributing to thymic immune damage (Yu et al., 2019). MG GroEL (HSP60) interacts with peripheral blood mononuclear cells (PBMCs) and DF-1 cells, inducing apoptosis, potentially through interaction with host Annexin A2 (Yu et al., 2019). Annexin A2 overexpression influences proliferation and apoptosis, linked to caspase activation and Bax/Bcl-2 ratios (Pérez-Sánchez et al., 2018). STAT3, a signaling molecule, regulates BCL2 and Bcl-xL expression (Xiu et al., 2016; Hart et al., 2011). These mechanisms highlight the role of oxidative stress and apoptosis in MG-induced immune pathology. In addition to directly inducing apoptosis and inflammation, MG employs more complex communal and metabolic strategies to ensure its persistence, such as biofilm formation and the production of cytotoxic metabolites.

### 2.4 Biofilm formation, glycerol metabolism, and hydrogen peroxide production

Beyond adhesion and direct cell damage, MG employs additional sophisticated strategies, including biofilm formation and specialized metabolism, to enhance its virulence and ensure persistent infection.

Biofilm formation is recognized as a critical virulence phenotype that contributes to the persistence of MG infections (Wang et al., 2017). *In vitro* studies have demonstrated that MG strains vary significantly in their biofilm-forming capacity. For instance, strains such as S6 and the vaccine strain 6/85 are prolific biofilm producers, while the attenuated vaccine strain ts-11 and the avirulent strain F36 show little to no biofilm formation (Chen et al., 2012). Biofilms provide a protected microenvironment, enhancing bacterial resistance to environmental stresses and antimicrobial agents, which likely facilitates chronic colonization in the host respiratory tract (Chen et al., 2012). The molecular basis of MG biofilm formation is complex, involving genes related to extracellular polysaccharide synthesis, lipoprotein production, translation, and metabolism (Wang et al., 2017).

Glycerol metabolism serves as a central energy pathway for MG, which lacks many biosynthetic capabilities. Glycerol is primarily imported via specific ABC transporters, such as MalF (re-annotated as part of the *golABC* operon) (Mahdizadeh et al., 2021). The import of glycerol is crucial; disruption of the *malF* gene not only perturbs global glycerol metabolism but also leads to a significant reduction in the pathogen’s ability to colonize and cause disease *in vivo*, underscoring its role in virulence (Mahdizadeh et al., 2021). Once inside the cell, glycerol is phosphorylated to glycerol-3-phosphate (G-3-P).

The oxidation of G-3-P is directly linked to hydrogen peroxide (H₂O₂) production, a major cytotoxic compound of mycoplasmas (Blötz and Stülke, 2017). This reaction is catalyzed by glycerol-3-phosphate oxidase, which generates H₂O₂ as a byproduct (Blötz and Stülke, 2017; Rice et al., 2001). H₂O₂ is a potent oxidizing agent that can damage host cell membranes (e.g., through lipid peroxidation) and intracellular components, contributing to the inflammation and tissue damage characteristic of MG infection, such as airsacculitis (Rice et al., 2001). Notably, the H₂O₂ production pathway is intrinsically tied to glycerol catabolism, making efficient glycerol uptake a prerequisite for the full expression of this virulence factor (Mahdizadeh et al., 2021; Blötz and Stülke, 2017).

In summary, these three mechanisms are interconnected: biofilm formation aids in persistence and protection, efficient glycerol metabolism fuels energy needs and provides the substrate, and the resultant H₂O₂ acts as a direct effector of host tissue damage. Together, they form an integrated system that enhances MG survival, colonization, and pathogenicity.

## 3 Vaccines

Vaccination is a key strategy for controlling MG in poultry. Available vaccines include live attenuated, inactivated (bacterins), and subunit vaccines, but protection is often partial or temporary (Ishfaq et al., 2020; Felice et al., 2020). Developing novel vaccines is crucial for effective MG control (Liu et al., 2024). Recent comprehensive reviews have detailed the progress and persistent challenges in this field, highlighting the ongoing need for vaccines that induce sterilizing immunity and are adapted to different host species and production systems (Mugunthan et al., 2023a).

### 3.1 Vaccine types

#### 3.1.1 Live attenuated vaccines

Live attenuated vaccines are widely used, providing durable immunity and reducing economic losses (Kanci Condello et al., 2020). Commercial strains include F (CEVAC MG F; Merial, now part of Boehringer Ingelheim, USA), K (VAXXON^®^ MG Live; Nisseiken Co., Ltd., Tokyo, Japan), ts-11 (VAXSAFE MG; Bioproperties Pty. Ltd., Australia), 6/85 strain (Zoetis, USA / Intervet, The Netherlands), and S6 (MG-Bac) (Mugunthan et al., 2023b). The 6/85, ts-11, and F strains are commercially approved, differing in protection, pathogenicity, and transmissibility (Ishfaq et al., 2020). Comparative genomic analyses of these attenuated strains have identified few common genetic changes, suggesting independent evolutionary paths to attenuation and highlighting the complex genetic basis of virulence in MG (Szczepanek et al., 2010). The F strain offers higher protection but is less attenuated and can cause disease in turkeys and under stress in chickens. In contrast, the ts-11 (a temperature-sensitive mutant) and 6/85 strains are safer but may offer a lower degree of protection (Miller et al., 2024). A critical safety concern for any live vaccine is the potential for reversion to virulence or difficulty in distinguishing vaccine strains from field isolates. Advanced molecular tools, such as whole-genome sequencing, have been employed to identify strain-specific genetic markers that can reliably differentiate the ts-11 vaccine from circulating field strains, which is crucial for effective disease surveillance and vaccination program monitoring (Kamathewatta et al., 2024). The ts-11 strain is a temperature-sensitive mutant administered via eye drop, colonizing the upper respiratory tract and inducing long-term immunity (Ishfaq et al., 2020). Its efficacy is dose-sensitive. The 6/85 strain induces lower serological responses but provides some protection (Ley et al., 1997; Bwala et al., 2018; Abd-el-Motelib and Kleven, 1993). The F strain offers higher protection but is less attenuated and can cause disease in turkeys (*Meleagris gallopavo*) (Abd-el-Motelib and Kleven, 1993; Leigh et al., 2020). The K strain, used mainly in Japan, shows effective protection in broilers and layers with minimal transmission risk (Bekő et al., 2020; Yadav et al., 2022b). Adhesion protein-based vaccines are promising candidates. GapA and CrmA are co-expressed in virulent Rlow and are essential for adhesion and virulence (Papazisi et al., 2002). The VaxSafe MG (TS-304) strain (Bioproperties Pty. Ltd., Australia), a GapA+ derivative of ts-11, colonizes the trachea and protects against virulent challenge (Kanci Condello et al., 2020). It is safe and effective at low doses (10^5^ CFU), with superior upper respiratory tract colonization compared to air sacs (Kanci Condello et al., 2020), likely due to GapA-mediated mucosal binding (Goh et al., 1998).

It is critical to recognize that vaccination strategies against MG for turkeys differ substantially from those for chickens. Live attenuated vaccine strains commonly used in chickens, such as the F strain and ts-11, are not suitable for turkeys; the F strain can retain pathogenicity in turkeys, while ts-11 exhibits poor colonization and provides limited protection in this species (Kanci et al., 2018; Kanci Condello et al., 2024). This disparity underscores the necessity for developing turkey-specific vaccines. Promising candidates, such as the GapA+ ts-304 strain (a derivative of ts-11), have been engineered to enhance colonization and have demonstrated both safety and significant protective efficacy against virulent MG challenge in turkeys (Kanci Condello et al., 2020). Addressing this species-specific gap remains a priority in MG vaccine development.

#### 3.1.2 Multi-epitope vaccines

Immunoinformatics enables prediction of potent T-cell and B-cell epitopes within antigenic proteins, facilitating epitope-based vaccine design (Mugunthan and Mani Chandra, 2021). Such vaccines target specific immune responses without causing hypersensitivity (Rosales et al., 2017). A multi-epitope vaccine targeting chicken TLR-2 and TLR-5 was constructed using epitopes from adhesion proteins (GapA, PlpA, Hlp3, CrmA) and VlhA (Mugunthan and Harish, 2021). Epitope vaccines offer advantages: reduced risk, engineered immunogenicity, chemical stability, cost-effectiveness, and safety (Mugunthan et al., 2023a). They represent promising candidates for MG control (Mugunthan and Harish, 2021).

Experimental validation of multi-epitope vaccines is an emerging area of research. One of the most advanced experimental studies developed a recombinant multi-epitope antigen (mEA) incorporating immunodominant epitopes from four key MG adhesion proteins (CrmA, GapA, Mgc2, and PvpA). Immunization of specific pathogen-free (SPF) chickens with this mEA vaccine elicited significantly higher levels of MG-specific antibodies compared to controls. Crucially, in a subsequent challenge experiment, birds vaccinated with the mEA antigen showed effective protection against tracheal mucosal damage caused by virulent MG infection, providing direct evidence of its protective potential (Mugunthan and Harish, 2021). In parallel, innovative production platforms are being validated. A separate study successfully expressed a plant-derived multi-epitopic peptide vaccine candidate containing conserved B-cell and T-cell epitopes from the same adhesion proteins in *Nicotiana benthamiana*. Administration of this plant-produced vaccine significantly boosted the production of epitope-specific IgY neutralizing antibodies in chickens, confirming the immunogenicity of the designed epitopes and the feasibility of the plant-based platform (Mugunthan et al., 2023b). These studies represent promising proof-of-concept steps; however, they also underscore that **c**omprehensive *in vivo* protection studies for purely computationally designed multi-epitope vaccines against MG are still limited, highlighting an important direction for future translational research.

#### 3.1.3 Inactivated vaccines

Inactivated vaccines are safer than live vaccines, eliminating reversion risk and inducing strong humoral immunity (Zhang et al., 2018). They reduce respiratory lesions and production losses (Ishfaq et al., 2020). INA (a hydrophobic alkylating agent) inactivates MG while preserving surface lipoproteins by targeting membrane lipid domains (Raviv et al., 2005). INA-inactivated MG induces strong antibody responses in chickens, suggesting its potential as a novel inactivating agent, though in vivo protection requires further validation (Atalla et al., 2015).

Inactivated (killed) vaccines, typically formulated as oil-emulsion bacterins, constitute a crucial tool for controlling MG, particularly in layer and breeder flocks where the use of live vaccines may be contraindicated. These vaccines are valued for their safety, as they eliminate the risk of reversion or horizontal transmission. Several commercial inactivated vaccines are available internationally. For instance, vaccines based on the R strain have been documented to reduce respiratory symptoms and egg production losses associated with MG (Wu et al., 2024). A commercially available inactivated vaccine containing the S6/85 strain (e.g., Nobilis MG Inac, Merck Animal Health) is also widely used. Beyond univalent formulations, multivalent inactivated vaccines that combine MG antigens with other pathogens offer a practical solution for comprehensive flock health management. Research indicates that pentavalent inactivated vaccines targeting Salmonella spp., MG, and *Mycoplasma synoviae* (MS) can provide effective protection against mycoplasma infections (Wu et al., 2024). This highlights a common trade-off: while extremely safe and stable, inactivated vaccines may require precise formulation and administration to achieve optimal protection levels. Future development efforts are exploring advanced inactivation methods, such as electron beam (eBeam) technology, aimed at developing multivalent vaccines with potentially broader cross-protective efficacy (Perera et al., 2025b). It should be noted that while effective, the protection conferred by inactivated vaccines may vary; for instance, one study reported a 66.6% protection rate based on air sac lesion scoring (Rh et al., 2018), which was somewhat lower than that offered by certain live attenuated vaccines under comparable conditions.

#### 3.1.4 Genetically engineered vaccines

Inactivated vaccines often fail to induce cellular immunity, while live vaccines carry reversion risks (Mase et al., 2010). Genetically engineered vaccines can overcome these limitations, offering stable phenotypes, no post-vaccination reactions, no virulence reversion, and limited horizontal spread (Hein et al., 2021). Recent advances are focused on rational antigen design and novel delivery platforms:

##### 3.1.4.1 Rational subunit vaccine design

A landmark study utilized knowledge of MG biology to develop a recombinant subunit vaccine containing the key adhesins GapA and CrmA, along with four early-phase-expressed variable lipoprotein hemagglutinins (VlhAs) from the virulent Rlow strain. In chicken trials, this multi-antigen vaccine, when formulated with the adjuvant CpG ODN, resulted in significant reductions in both MG recovery and tracheal pathology after challenge, demonstrating the promise of rationally designed, safe subunit vaccines (Miller et al., 2024).

##### 3.1.4.2 Vectored vaccines

Bivalent vaccines reduce vaccination pressure and enable combined antigen delivery (Du et al., 2017). Adenovirus vectors, which infect diverse host cells and achieve high titers (Yamamoto et al., 2017), have been used to express heterologous proteins (Cabrera-Mora et al., 2016). A recombinant adenovirus vaccine (pBH-S1-TM-1-EGFP, a research vaccine) expressing an MG antigen elicited high antibody titers and protection without causing significant damage in chickens (Zhang et al., 2018), demonstrating potential as a bivalent vaccine against MG and infectious bronchitis virus (IBV) (Zhang et al., 2018). Recombinant adenovirus safety profiles are comparable to fowlpox-vectored vaccines (research vaccines) (Zhang et al., 2010; Leigh et al., 2013).

In addition to adenovirus vectors, recombinant fowlpox virus (FPV) vectors represent another prominent and commercially available platform for MG vaccination (Wu et al., 2024; Zhang et al., 2010). A widely recognized example is the VECTORMUNE® FP-MG vaccine (Biomune Company, USA), a live recombinant fowlpox virus engineered to express key protective antigens of MG, namely the 40 k and *mgc* genes (Zhang et al., 2010). This vectored vaccine is designed to provide dual protection against both fowlpox and MG infections. A dedicated safety assessment in specific-pathogen-free chickens confirmed that the VECTORMUNE® FP-MG vaccine exhibits a high level of safety, with no significant clinical signs or adverse effects on air sacs upon vaccination, and demonstrates genetic stability without horizontal transmission (Zhang et al., 2010). Beyond FPV, other viral vectors such as herpesvirus of turkey (HVT) are also being actively investigated as platforms for delivering MG antigens, highlighting the continued expansion of vectored vaccine strategies in poultry health management (Wu et al., 2024).

### 3.2 Factors influencing vaccine efficacy

Vaccine efficacy depends on delivery method, administration route, adjuvants, dose, vaccination-sampling interval, host susceptibility, and bird genetics/physiology (Feberwee et al., 2022). Delivery methods include spray, eye drop, and drinking water; spray is cost-effective but eye drop often induces stronger immune responses (Leigh et al., 2018; Evans et al., 2015). Adjuvants enhance immunogenicity (Ji et al., 2025). Host factors significantly affect the minimum effective dose (Feberwee et al., 2022; Whithear et al., 1990). An often-overlooked factor is the interaction between vaccination and antimicrobial therapy. A transcriptomic study revealed that while administration of tylosin (a common macrolide antibiotic) after vaccination with Vaxsafe MG ts-304 reduced the duration of immunity, the initial protective immunity still lasted for at least 22 weeks post-vaccination. The study confirmed that vaccination induced a rapid and effective secondary immune response that was fundamentally different from the dysregulated inflammatory response seen in unvaccinated birds upon challenge (Mugunthan et al., 2023b).

### 3.3 Vaccination limitations and novel vaccine development

Current commercial vaccines (live attenuated and inactivated) have limitations: live vaccines may cause side effects or revert, while inactivated vaccines require repeated administration and are costly (Ishfaq et al., 2020). During outbreaks, these vaccines may not control infection effectively (Fatideh et al., 2022). MG’s ability to invade non-phagocytic cells, regulate host microRNAs, modulate inflammation, and impair macrophage/lymphocyte function, coupled with antigenic variability and vaccine instability, necessitates novel vaccine development. Research should focus on MG’s immune evasion mechanisms to design more effective, affordable, and stable vaccines (Table 2).

**Table 2:** Overview of vaccine types for MG.

Vaccine type	Examples	Key advantages	Key limitations
Live attenuated	F strain, ts-11, 6/85, VaxSafe MG (TS-304)	Induces strong cellular and local mucosal immunity; longer duration of protection.	Potential for residual pathogenicity (F strain); can interfere with serology; dose-sensitive.
Inactivated (Bacterin)	Oil-emulsion vaccines, INA-inactivated vaccine	Safe, no risk of replication or spread; stable.	Primarily induces humoral immunity; requires injection; may need booster; shorter immunity.
Multi-epitope	Computationally designed chimeric antigens	Safe, specific, chemically stable; can target multiple antigens.	Still largely in experimental stages; may require potent adjuvants; efficacy in field trials needed.
Recombinant viral vector	Adenovirus-pBH-S1-TM-1-EGFP, Fowlpox-vectored	Safe; can induce both humoral and cellular immunity; potential for bivalent vaccines.	Pre-existing immunity to the vector might reduce efficacy.
Recombinant yeast	EBY100/pYD1-TM1 (Oral vaccine)	Safe (GRAS organism); easy oral administration; induces mucosal and systemic immunity; cost-effective.	New technology; long-term protection and field efficacy data pending.

To overcome these limitations, next-generation platforms are under investigation. Electron beam (eBeam) inactivation technology is being explored as an alternative to traditional chemical methods for producing killed vaccines (Perera et al., 2025a). eBeam irradiation effectively disrupts pathogen nucleic acids while potentially better preserving conformational antigenic epitopes (Praveen et al., 2013). Research initiatives, such as a USDA-funded project, aim to leverage this technology to develop a multivalent vaccine targeting multiple MG strains, which may offer broader cross-protection. Furthermore, combined vaccine formulations are gaining attention for their potential to simplify immunization protocols and enhance overall flock health. Studies have explored the preparation and efficacy of a recombinant vaccine targeting both MG and *Mycoplasma synoviae*. Another innovative approach involves integrating vaccination with immunomodulation; for instance, a study demonstrated that a multivalent eBeam-killed vaccine administered alongside a probiotic program significantly reduced the incidence of bacterial chondronecrosis with osteomyelitis in a challenge model, suggesting a synergistic effect for complex disease prevention (Anthney et al., 2025).

### 3.4 Advances in genomics and reverse vaccinology

Recent advances in high-throughput sequencing and bioinformatics have accelerated the discovery of novel vaccine targets through reverse vaccinology. Whole-genome sequencing of over 150 MG isolates from Asia, Europe, and North America identified conserved surface-exposed proteins absent in non-pathogenic mycoplasmas, including hypothetical proteins MG_219 and MG_492 (Mugunthan and Harish, 2021).

Pan-genome analysis revealed a core genome of ~680 genes shared across all isolates, with accessory genes enriched in mobile elements and *vlhA*-like repeats, reflecting horizontal gene transfer and adaptive evolution (Pflaum et al., 2018). Machine learning algorithms were applied to predict antigenicity, subcellular localization, and essentiality, narrowing down potential candidates for experimental validation.

Notably, CRISPR-Cas screening in infected DF-1 cells (chicken fibroblasts) identified several host dependency factors, including integrin α5β1 and CD44, which interact with MG adhesins (Ipoutcha et al., 2024). Targeting these host-pathogen interfaces offers a new avenue for intervention, either through blocking peptides or monoclonal antibodies.

Additionally, single-cell RNA sequencing (scRNA-seq) of MG-infected airway epithelia uncovered distinct cellular subsets with differential susceptibility, revealing that ciliated cells express higher levels of *GapA* receptors than goblet or basal cells (Limsatanun et al., 2022). This spatial resolution informs targeted delivery strategies for next-generation vaccines.

## 4 Challenges and future directions

Despite decades of research and the availability of various vaccine types, the effective control of *Mycoplasma gallisepticum* (MG) remains a formidable challenge in global poultry production. The persistent difficulties stem from the intricate interplay between the pathogen’s sophisticated virulence mechanisms and the limitations of current intervention tools. This section delineates the core challenges and outlines future research directions that are intrinsically guided by an in-depth understanding of MG pathogenesis (Mugunthan et al., 2023a; Wu et al., 2024).

### 4.1 Current challenges

#### 4.1.1 Limitations of current vaccines

The primary obstacle in MG management is the suboptimal performance of existing vaccines, a direct consequence of the pathogen’s immune evasion strategies. Live attenuated vaccines (e.g., F, ts-11, 6/85), while inducing durable immunity, carry risks of residual pathogenicity, reversion, or interference with disease surveillance. Inactivated (killed) bacterins, though safe, often fail to elicit robust mucosal or cellular immunity, leading to incomplete protection that does not prevent colonization or shedding (Wu et al., 2024). Crucially, no current vaccine provides sterilizing immunity. This allows for subclinical infections and intermittent shedding in vaccinated flocks, perpetuating silent transmission within and between farms (Mugunthan et al., 2023a). The fundamental issue is that many traditional vaccines were developed empirically without fully targeting the molecular basis of MG’s persistence, such as its antigenic variation and immune modulation capabilities.

#### 4.1.2 The diagnostic bottleneck: inability to link infection to pathogenic mechanism

Effective surveillance and vaccine efficacy evaluation are severely hampered by diagnostic shortcomings. Standard serological assays (e.g., serum plate agglutination, ELISA) cannot differentiate between antibodies induced by vaccination and those resulting from natural infection (DIVA principle). This complicates eradication programs in vaccinated populations. More importantly, these methods lack the resolution to connect an infection to specific virulent strains or mechanisms. They cannot identify which variant of a key antigenic protein (e.g., a specific VlhA) is circulating, hindering our ability to track strain evolution in relation to vaccine pressure or to understand outbreak dynamics at a mechanistic level.

To overcome the limitations of serology, molecular assays, particularly real-time quantitative PCR (qPCR), have become the cornerstone of MG diagnostics. Studies have directly demonstrated the superior sensitivity of molecular methods (Yadav et al., 2024); for instance, qPCR on cultured broth showed a positivity rate of 89.0%, significantly higher than the 34.5% achieved by conventional culturing techniques. Moreover, qPCR targeting specific housekeeping genes like mgc2 has been shown to be highly effective, with detection rates reaching 69%. This method provides earlier detection than antibody-based tests and allows for the quantification of pathogen load. However, a standard positive qPCR result only confirms the presence of MG nucleic acid and cannot by itself differentiate between viable and non-viable organisms, specific strain types, or virulence markers. This necessitates coupling qPCR with sequencing. For strain differentiation and phylogenetic analysis, sequencing of qPCR amplicons (e.g., of the mgc2 or atpG genes) is employed to distinguish isolates into different clades with high discriminatory power. While this combined approach (“qPCR coupled with sequencing”) provides definitive identification and can link an infection to specific bacterial genotypes, the sequencing step remains relatively specialized, time-consuming, and costly, limiting its routine application in all diagnostic settings (Al-Baqir et al., 2023).

### 4.2 Future research directions

To address these persistent challenges, future research must pivot from empirical approaches to mechanism-driven strategies, focusing on the development of next-generation vaccines and precision diagnostics. Future efforts must leverage insights from MG pathogenesis to develop next-generation tools for precise detection and immunization.

#### 4.2.1 Rational design of next-generation vaccines

The future of MG vaccinology lies in rational design based on a deep understanding of virulence factors. Promising strategies include:

##### 4.2.1.1 Multi-antigen subunit vaccines

Combining key conserved adhesins (e.g., GapA, CrmA) with strategically selected variable antigens (e.g., specific VlhA variants) to broaden protection and counteract immune escape. A seminal 2024 study demonstrated the efficacy of a subunit vaccine containing GapA, CrmA, and four early-expressed VlhAs, which, when paired with a CpG ODN adjuvant, significantly reduced tracheal pathology and MG recovery in challenged chickens (Miller et al., 2024). Research into plant-derived expression systems for multi-epitope vaccines also presents a cost-effective and scalable alternative (Mugunthan et al., 2023a; Wu et al., 2024).

##### 4.2.1.2 Focus on mucosal immunity

Building on the mucosal immunity elicited by current live attenuated vaccines, future strategies aim to induce a more potent, rapid, and durable local defense. The goal is not merely to replicate but to qualitatively enhance mucosal immune responses. Key future directions include: (1) employing novel mucosal adjuvants and delivery systems designed to overcome mucosal barriers and enhance antigen uptake, thereby eliciting stronger secretory IgA responses and tissue-resident memory T cell formation at the respiratory tract (Park et al., 2024); (2) applying systems vaccinology approaches to rationally design vaccine platforms and adjuvants that optimally activate protective local immune pathways, moving beyond empirical design (Vilander et al., 2024); and (3) developing multivalent mucosal vaccines that can simultaneously target multiple pathogens or strains, offering broader protection at the primary site of infection (Ogonczyk-Makowska et al., 2024). Insights from other mucosal pathogens also highlight innovative platforms, such as engineered proteins that ‘hitchhike’ across the mucosal barrier using endogenous transport mechanisms, dramatically enhancing antigen delivery and subsequent antibody titers in mucosal tissues (Hartwell et al., 2022). By focusing on these advanced strategies, next-generation MG vaccines could achieve superior local immune stimulation, leading to more effective and sustained prevention of initial colonization.

#### 4.2.2 Development of mechanism-aware diagnostic tools

Advanced diagnostics must evolve from merely detecting infection to characterizing the pathogen’s mechanistic profile.

##### 4.2.2.1 DIVA-compliant assays

To address the critical need for differentiating infected from vaccinated animals (DIVA), both molecular-based and protein-based strategies are under development. While the development of assays using recombinant proteins or peptides absent in vaccine strains remains a promising approach for future serological DIVA tests, polymerase chain reaction (PCR)-based molecular assays already provide a powerful and widely implemented solution to overcome key limitations of traditional serology. Compared to antibody detection methods like ELISA, which can be influenced by cross-reactions and delayed seroconversion, PCR offers superior sensitivity and specificity for the direct detection of MG DNA, enabling earlier diagnosis (Yadav et al., 2022a). More importantly, advanced molecular techniques can be designed to achieve differentiation. Vaccine strain-specific PCR assays have been successfully applied in field surveillance. For instance, a study utilizing such an assay found that among MG PCR-positive samples from commercial flocks, a significant proportion (e.g., 56.1% of tracheal swabs) were identified as originating from the live F-strain vaccine, directly demonstrating the ability to distinguish vaccination from field strain infection in a diagnostic setting (Amorim et al., 2024). Furthermore, innovative surveillance methods such as monitoring viral nucleic acids in poultry dust have shown potential as a population-level screening tool to non-invasively track vaccine uptake and pathogen status in flocks (Assen et al., 2022). These molecular tools collectively provide diagnostic laboratories with practical means to conduct effective surveillance in vaccinated populations, complementing the ongoing pursuit of ideal serological DIVA tests.

##### 4.2.2.2 Genomic and proteomic surveillance

Widespread application of whole-genome sequencing will allow for high-resolution tracking of field strains, monitoring genetic drift in key virulence genes, and establishing direct links between genetic markers and clinical outcomes. This can guide vaccine antigen updates and outbreak investigations.

#### 4.2.3 Integration of pathogenetic insights into control strategies

Finally, control strategies must integrate these advanced tools. Understanding how MG factors like biofilm formation or glycerol metabolism contribute to persistence can inform management practices. The synergy between mechanism-based vaccines and precision diagnostics will create a feedback loop: diagnostics will monitor the efficacy of new vaccines against evolving strains, while vaccine pressure will be tracked to anticipate epidemiological shifts. This integrated, knowledge-driven approach is essential for moving from partial control toward sustainable mitigation and potential eradication of MG in poultry populations.

## 5 Conclusion

This review synthesizes the current understanding of MG pathogenesis and vaccine development. A central theme that emerges is the intimate and reciprocal relationship between virulence mechanisms and immunization strategies. The pathogen’s sophisticated tactics—including adhesion via GapA/CrmA, antigenic variation through VlhA/pMGA, immune modulation, and biofilm formation—directly define the challenges faced by current vaccines, such as incomplete protection and the inability to induce sterilizing immunity.

Conversely, advances in elucidating these pathogenic pathways are illuminating the path toward next-generation solutions. The future of MG control lies in leveraging this mechanistic knowledge to engineer more precise tools. This includes the rational design of multi-antigen vaccines targeting conserved and variable epitopes, the development of novel vectored and platform technologies to induce robust mucosal immunity, and the creation of mechanism-aware diagnostics capable of differentiating infected from vaccinated animals and tracking strain evolution. Moving forward, an integrated approach that continuously translates insights from pathogenesis research into refined vaccines and diagnostics will be paramount for achieving sustainable control and mitigating the significant economic burden imposed by MG on global poultry production.
